# Oxidative stress disrupts the cytoskeleton of spinal motor neurons

**DOI:** 10.1002/brb3.2870

**Published:** 2022-12-29

**Authors:** Jian Chen, Tianyu Chen, Yeyang Wang, Juanjuan Meng, Guangjiao Tan, Qiurong Zhao, Shilong Feng, Lixin Xu, Qinqin Pei

**Affiliations:** ^1^ Department of Orthopedics Chongqing University Three Gorges Hospital Chongqing China; ^2^ Chongqing Municipality Clinical Research Center for Geriatric Diseases Chongqing University Three Gorges Hospital Chongqing China; ^3^ Central Laboratory Chongqing University Three Gorges Hospital Chongqing China; ^4^ School of Graduate North Sichuan Medical College Nanchong China; ^5^ Department of Orthopedics The Third Affiliated Hospital of Southern Medical University Guangzhou China; ^6^ Department of Spinal Surgery Guangdong Second Provincial General Hospital Guangzhou China

**Keywords:** cytoskeleton, N‐acetyl‐l‐cysteine, oxidative stress, spinal cord injury, spinal motor neurons

## Abstract

**Background and aim:**

Traumatic spinal cord injury (SCI) is a common and devastating central nervous disease, the treatment of which faces many challenges to the medical community and society as a whole. Treatment measures based on oxidative stress of spinal motor neurons during SCI are expected to help restore biological functions of neurons under injury conditions. However, to date, there are no systematic reports regarding oxidative stress on spinal motor neuron injury. Our aim is to better understand and explain the influences and mechanisms of oxidative stress on spinal motor neurons during SCI.

**Methods:**

We first exposed VSC4.1 motor neurons to hydrogen peroxide (H_2_O_2_) and evaluated the effects on cell viability, morphology, cycling, and apoptosis, with an emphasis on the changes to the cytoskeleton and the effect of *N*‐acetyl‐l‐cysteine (NAC) on these changes. Then, we investigated the effects of NAC on these cytoskeletal changes in vitro and in vivo.

**Results:**

We found that H_2_O_2_ caused severe damage to the normal cytoskeleton, leading to a reduction in neurite length and number, rearrangement of the actin cytoskeleton, and disorder of the microtubules and neurofilaments in VSC4.1. Importantly, NAC attenuated the oxidative damage of spinal motor neurons in vitro and in vivo, promoting the recovery of hindlimb motor ability in mice with SCI at the early stage of injury.

**Conclusion:**

This study shows that oxidative stress plays an important role in the cytoskeleton destruction of spinal motor neurons in SCI, and treatment of SCI on this basis is a promising strategy. These findings will help to elucidate the role of oxidative stress in spinal motor neuron injury in SCI and provide references for further research into the study of the pathology and underlying mechanism of SCI.

## Graphical abstract

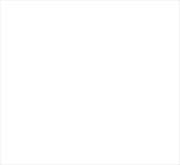

## 1 INTRODUCTION

Traumatic spinal cord injury (SCI) is a clinically common and devastating central nervous disease, which occurs during external physical shocks, such as falls, violence, and sports‐related injuries (Ahuja et al., 2017; El Masri et al., 2011). It not only brings great physical and emotional burdens to the patients and their families but also puts enormous pressure on the clinical management and society as a whole (GBD 2016 Neurology Collaborators, 2019; Lee et al., 2014; Rubiano et al., 2015). In the past few decades, exciting progress has been made in understanding the pathophysiology of SCI, which has resulted in better treatment paradigms (Cadotte & Fehlings, 2013; David et al., 2019; Fouad et al., 2021; Sofroniew, 2018; Zawadzka et al., 2021). However, the therapeutic effect on clinical patients has not yet achieved satisfactory results and a huge global challenge for the treatment of SCI still exists (Filli & Schwab, 2012; Selvarajah et al., 2015; Hejrati & Fehlings, 2021; Squair et al., 2021).

Spinal motor neuron injury is a primary cause of permanent disability in SCI patients, and increasing numbers of studies have been conducted on the intervention of damaged and residual motor neurons, hoping to promote the recovery of some motor functions, with varying results (Grumbles & Thomas, 2017; Li et al., 2019; Wang et al., 2021).

Oxidative stress, defined as the imbalance between oxidants and antioxidants, occurs in the initial stage of SCI and evolves in the following months, triggering a series of molecular and cellular events (Azbill et al., 1997; von Leden et al., 2017; Sies & Jones, 2020). Unfortunately, during this process, the spinal motor neurons are more vulnerable to damage due to their high energy requirements and complex morphology, resulting in continuous deterioration of neurological function (Grossman et al., 2001; Xu et al., 2005; Zrzavy et al., 2021). Treatment based on an oxidative injury of spinal motor neurons is a promising strategy for SCI, and combined with other therapeutic strategies, it may better promote the repair of neurons after SCI. Moreover, it is well known that in‐depth research and a comprehensive understanding of pathological changes are prerequisites for formulating targeted and efficient disease treatment strategies. More, oxidative damage itself is multifaceted, and the resultant injury to spinal motor neurons during and after SCI is varied. Therefore, it is necessary to conduct more detailed studies regarding oxidative stress itself and its effects on spinal motor neurons, as well as the effects of treatment targeting it in SCI.

In this study, we exposed VSC4.1 spinal motor neuron cell lines to hydrogen peroxide (H_2_O_2_) at different concentrations and times and evaluated the effects on cell morphology, cycling, and apoptosis. At the same time, the destructive effects on the cytoskeleton were analyzed and discussed. Furthermore, the effect of *N*‐acetyl‐l‐cysteine (NAC) on these cytoskeletal changes was investigated both in vitro and in vivo. Our aim is to better understand the multifaceted changes of motor neuron function caused by oxidative damage in vitro, so as to increase our knowledge in this field, and provide a theoretical basis and reference for further research into slowing disease progression and providing new treatment strategies for SCI.

## 2 MATERIALS AND METHODS

### 2.1 Animals and model establishment

C57BL/6 female mice (12 weeks, 18−22 g) were used in this study. All animal experiments were approved by the Animal Care and Use Ethics Committee of Chongqing University Three Gorges Hospital and strictly adhered to the guidelines for the ethical treatment of animals.

All mice were randomly divided into four groups: sham operation (Sham, *n* = 8), SCI operation (SCI, *n* = 8), sham operation with NAC treatment (Sham+NAC, *n* = 8), and SCI operation with NAC treatment (SCI+NAC, *n* = 8). Mice in the four groups underwent laminectomy at the T9–T11 levels to expose the spinal cord. For the mice in the SCI and SCI+NAC groups, the modified Allen method was used to establish SCI at the T10 level. Penicillin (100 mg/kg) was injected intraperitoneally for 3 consecutive days to prevent infection at the incision site, and the bladder was massaged twice daily to assist in urination. The padding material was changed daily to maintain sanitary conditions in the cage. NAC (150 mg/kg, IA0050, Solarbio, Beijing, China) was intraperitoneally injected into mice in the SCI+NAC and Sham+NAC groups once a day. After 3 days, all the mice were sacrificed by cervical dislocation, and the spinal cords were excised for histological assessment.

### 2.2 Staining of spinal cord tissue sections

Three days after surgery, the mice were sacrificed with a lethal level of isoflurane. The spinal cord segments containing the injured area were removed after perfusion with phosphate‐buffered saline (PBS) and 4% paraformaldehyde. Tissues were stored in 4% polyformaldehyde solution overnight at 4°C, embedded in paraffin, and sectioned in cross sections (2 μm). The paraffin sections in each group were stained with hematoxylin and eosin staining to observe histological changes. Immunofluorescence staining of tissue sections was as follows: Four sections for each mouse were first washed with PBS, then incubated in 0.1% Triton X‐100 for 5 min, blocked with 10% nonspecific fetal bovine serum for 1 h, and incubated overnight at 4°C with primary antibody to neuronal marker microtubule‐associated protein 2 (MAP‐2; ab32454, 1:50, Abcam, Cambridge, UK). The sections were then incubated with secondary antibodies Alexa Fluor 647 (ab150107, Abcam) and Alexa Fluor 488 (#4412, Cell Signaling Technology, Danvers, MA, USA) for 1 h at room temperature. Transferase dUTP nick end labeling (TUNEL) assay solution was added according to the instructions of the TUNEL apoptosis detection reagent (C1090, Beyotime Biotechnology Institute, Shanghai, China). Finally, the nuclei were counterstained by 4′,6‐diamidino‐2‐phenylindole (DAPI; C0065, Solarbio), and cells were observed with fluorescence microscopy (Olympus BX63, Tokyo, Japan).

### 2.3 Assessments of motor function

Motor function scores in the four groups of experimental mice were evaluated with hindlimb motor function evaluated through the Basso, Beattie, and Bresnahan (BBB) scoring (Basso et al., 1995) in open areas by two independent researchers. The mice were evaluated for the first time after surgery, and then daily for 7 days. The results were recorded on a scale of 0−21 based on performance.

### 2.4 Cell culture and treatment

The motor neuron cell line VSC4.1 was used in this study (Hammond et al., 1986; Crawford et al., 1992). VSC4.1 cells (Beijing BTRAK Institute of Biotechnology, Beijing, China) were cultured in Dulbecco's modified hyperglucose medium (DMEM‐HG; HyClone Laboratories, LLC, Logan, UT, USA) with 100 U/ml penicillin and streptomycin (Beyotime Biotechnology Institute, Shanghai) supplemented with 10% fetal bovine serum (Thermo Fisher Scientific, Waltham, MA, USA) at 37°C and 5% CO_2_. Staining was performed with the neuronal marker MAP‐2 to validate VSC4.1 cells (Crawford et al., 1992). To induce oxidative damage, VSC4.1 cells were exposed to H_2_O_2_ at different concentrations for different times with or without NAC, and the subsequent experiments were performed.

### 2.5 Cell viability analysis

The cell counting kit 8 (CCK‐8) was used to measure cell viability in our experiment. In brief, VSC4.1 cells were digested by trypsin and inoculated in 96‐well plates, incubated with different concentrations (0, 100−1400 μM) of H_2_O_2_ at 37°C at various time points (0, 2, 4, 6, 8, 12, and 24 h) with four parallel wells in each treatment. The CCK‐8 staining kit (#C0041, Beyotime Institute of Biotechnology) was used following the manufacturer's instructions after all processing was completed. Briefly, the medium in each well was replaced with a 100 μl mixed solution of 10 μl CCK‐8 test solution and 90 μl fresh medium and incubated for 2 h at 37°C. The optical density of each well was measured at 450 nm using a microplate reader (Molecular Devices).

### 2.6 Western blot

VSC4.1 cells in each group were harvested in RIPA buffer, and the lysates were homogenized. The homogenized samples were gently rotated in a cold chamber for 20 min and then centrifuged at 15,000 *g* for 10 min. The BCA assay was used for the quantification of protein concentration. After, 10 μg of denatured protein from each sample was loaded onto a prefabricated protein gel (Bio‐Rad) for SDS–PAGE and then transferred to a nitrocellulose membrane. Next, 5% nonfat dry milk in Tris‐buffered saline containing 0.1% Tween 20 was used to block the membranes for 2 h at room temperature, after which they were incubated with MAP‐2 (ab32454, 1:1000, Abcam), α‐tubulin (#3873, 1:1000, Cell Signaling Technology), β‐tubulin (#2128, 1:1000, Cell Signaling Technology), neurofilament heavy (NF‐H) (#2836, 1:1000, Cell Signaling Technology), P21 (ab109520, 1:5000, Abcam), Bcl‐2 (ab59348, 1:1000, Abcam), Bax (#2772, 1:1000, Cell Signaling Technology), β‐actin (1:5000; Sigma‐Aldrich, USA), and GAPDH (#5174, 1:1000, Cell Signaling Technology) for 16 h at 4°C. Membranes were washed with Tris‐buffered saline containing 0.1% Tween 20 for four times (10 min/cycle), after which the antibodies were conjugated with ECL rabbit or mouse IgG HRP‐linked (Cytiva, USA) for 1 h at room temperature. The density of the protein bands was measured by ImageJ software (National Institutes of Health, Bethesda, MD, USA).

### 2.7 Immunocytochemistry

After the H_2_O_2_ exposure, VSC4.1 cells were rinsed with PBS, fixed with 4% paraformaldehyde (G1101, Servicebio, Wuhan, China), and permeabilized with 0.3% Triton X‐100 (T8200, Solarbio) for 15 min. Nonspecific binding was blocked with 5% BSA Blocking Buffer (SW3015, Solarbio). Next, VSC4.1 cells were incubated with primary antibodies MAP‐2 (ab32454, 1:50, Abcam), NF‐H (#2836, 1:100, Cell Signaling Technology), α‐tubulin (#3873, 1:1000, Cell Signaling Technology), and β‐tubulin (#2128, 1:100, Cell Signaling Technology) overnight at 4°C. Cells were then incubated with secondary antibodies Alexa Fluor 647 (ab150107, Abcam, UK) and Alexa Fluor 488 (#4412, Cell Signaling Technology) for 1 h at room temperature and counterstained with DAPI (C0065, Solarbio) to visualize the nuclei. Finally, the cells were imaged under a fluorescence microscope (Olympus BX63), and the fluorescence intensity was quantified by ImageJ software (National Institutes of Health).

### 2.8 Cell morphology observation

In our experiments, following hematoxylin staining, the general morphology of VSC4.1 cells in the different groups was observed under a light microscope (Olympus B61, Tokyo, Japan). To assess changes in the microfilament cytoskeleton, rhodamine‐phalloidin (CA1610, Solarbio) and DAPI staining were used to visualize F‐actin and nuclei, respectively. Images were acquired with a confocal microscope (Leica, Wetzlar, Germany). Neurite length evaluation of VSC4.1 cells was conducted using ImageJ software (National Institutes of Health).

### 2.9 Flow cytometry for analysis of the cell cycle

Analysis of the cell cycle in VSC4.1 cells was assessed with propidium iodide (PI) staining. Cells were washed once with ice‐cold PBS, resuspended in ice‐cold PBS, and incubated with PI and RNaseA for 30 min at room temperature. Finally, the cell cycle status was analyzed with a flow cytometer (Beckman, CytoFLEX).

### 2.10 Flow cytometry for analysis of apoptosis

Cell membrane integrity was detected by flow cytometry using Annexin V, which binds phosphatidylserine, to further evaluate the apoptosis of VSC4.1 cells after H_2_O_2_ exposure. VSC4.1 cells were placed in 6‐well plates and then incubated with H_2_O_2_ at different concentrations (0, 100−800 μM). An Annexin V‐FITC Apoptosis Detection Kit (Beijing Solarbio Science & Technology Co., Ltd., Beijing, China) was used according to manufacturer's instructions and the cellular apoptosis rate was analyzed using flow cytometry (Beckman, CytoFLEX). The data were analyzed and apoptosis was assessed in living cells, identified as Annexin V/PI negative cells. Annexin V^−^ PI^+^ (upper left) indicates the necrotic cells. The apoptosis rate was calculated as the sum of the proportion of early apoptosis (Annexin V^+^ PI^−^, upper right) cells and late apoptotic cells (Annexin V^+^ PI^+^, lower right).

### 2.11 Detection of intracellular reactive oxygen species (ROS)

We assessed intracellular reactive oxygen species (ROS) production by measuring ROS concentrations. VSC4.1 cells were inoculated in 96‐well culture plates and treated with H_2_O_2_ (0, 100−800 μM) for 2 h, and with or without NAC (2 mM) for 2 h. After that, the cells were washed once with PBS and then incubated in serum‐free DMEM containing DCFH‐DA for 20 min at 37°C according to the instructions of an ROS assay kit (#BC01010, Bioss, Beijing, China). After that, cells were washed with PBS. Finally, images were obtained by fluorescence microscopy (Olympus BX63).

### 2.12 Statistical analysis

GraphPad Prism (GraphPad software, Version 8.3, CA, USA) was used for statistical analysis. One‐way or two‐way analysis of variance was performed followed by Tukey's multiple comparisons test. All experiments in this study were performed at least three biological replicates. When the *p* values were ≤.05, the differences were considered statistically significant. Data are expressed as means ± standard error of mean (SEM).

## 3 RESULTS

### 3.1 H_2_O_2_ reduces the viability of VSC4.1 cells

**FIGURE 1:**
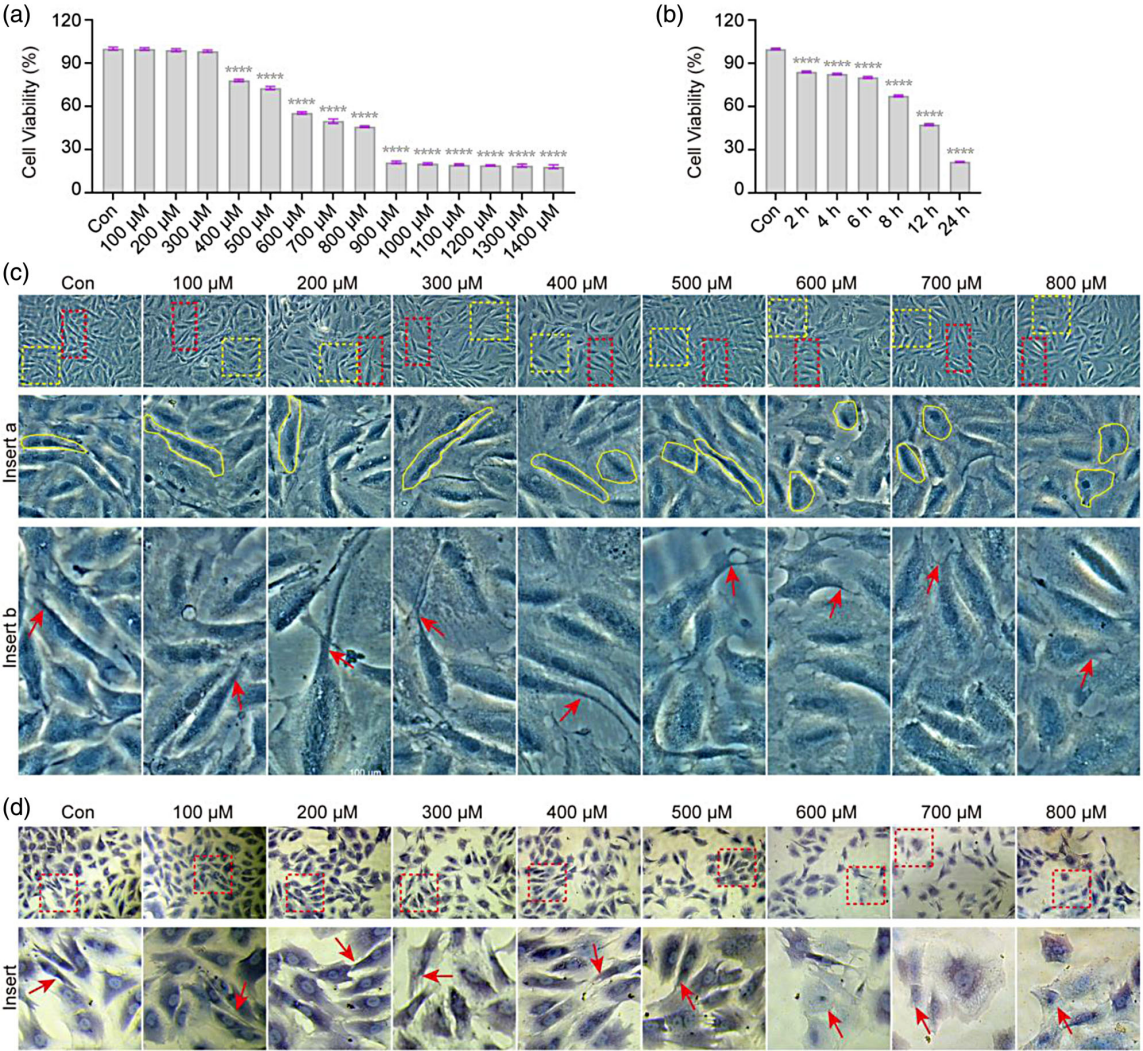
**H_2_O_2_ changes morphology of VSC4.1 cells**. (a and b) VSC4.1 cells were treated with hydrogen peroxide (H_2_O_2_) using a concentration gradient for 2 h and time gradient at 400 μM. Cell viabilities analysis using the cell counting kit 8 (CCK‐8) revealed that the viability of VSC4.1 cells reduced in a dose‐ (a) and time‐dependent (b) manner. (c and d) VSC4.1 cells were treated at the indicated concentrations of H_2_O_2_ and morphological changes were observed. (c) Representative images under a light microscope showed altered original cell shape (yellow curve) and shortened or even disappeared neurites (red arrow →) in VSC4.1 cells treated with H_2_O_2_ (from 400 to 800 μM). Scale bars: 100 μm. (d) Representative images of VSC4.1 cells stained with hematoxylin under a light microscope showed shortened or even disappeared neurites (red arrow →) in VSC4.1 cells treated with H_2_O_2_ (from 600 to 800 μM). Scale bars: 100 μm. One‐way analysis of variance (ANOVA) followed by Tukey's multiple comparisons test. Data are presented as mean ± standard error of mean (SEM). Compared with the control group (Con), ****p < .0001

In order to analyze the injury associated with oxidative stress in spinal motor neurons in vitro, the motor neuron cell line VSC4.1 derived from the fusion of rat embryonic spinal cord ventral neuron cells with N18TG2 neuroblastoma cells was selected in this study (Hammond et al., 1986; Crawford et al., 1992; Smith et al., 1994). H_2_O_2_, an oxidant commonly used in the establishment of oxidative stress cell models in vitro, was used as the inducer (Sies & Jones, 2020). We first assessed the viability of VSC4.1 cells exposed to H_2_O_2_ with varying concentrations (0, 100−1400 μM) for 2 h and at 400 μM for different times (0, 2, 4, 6, 8, 12, and 24 h) using the CCK‐8 assay. As shown in Figure 1a, concentrations from 100 to 300 μM caused no significant changes in cell viability; however, at 400 μM, the viability began to decrease in a dose‐dependent manner. Furthermore, when treated with the same concentration of 400 μM, the cell viability decreased by 18% at 2 h, and a significantly reduced cell viability over time was observed in VSC4.1 cells (*p* < .0001; Figure 1b). These results show that H_2_O_2_ reduces the viability of VSC4.1 cells in a dose‐ and time‐dependent manner.

### 3.2 H_2_O_2_ changes the morphology of VSC4.1 cells

The polarized morphology of neurons is a functionally important characteristic (Smith et al., 1994). Therefore, the effects of H_2_O_2_ on VSC4.1 cell morphology were next determined. We first observed the general morphological changes of VSC4.1 cells using a light microscope after exposure to different concentrations of H_2_O_2_. Normal VSC4.1 cells showed a fibroid shape, protruding in the middle, and elongating at both ends, with no significant morphological changes observed after H_2_O_2_ exposure at 100–300 μM. However, when the concentration increased to 400 μM, the original shape of the cells became altered, granular changes appeared, and the nucleus became condensed and deviated from the cell edge (Figure 1c). A similar morphological change trend was also observed by hematoxylin staining (Figure 1d). It is well documented that neuritides are key to the functioning of neurons, and that altered neurite shape, size, and number are associated with a variety of central nervous diseases (Donato et al., 2019; Kennedy & Hanus, 2019). Therefore, we further analyzed the effects of H_2_O_2_ on VSC4.1 cell neurites. When the concentration of H_2_O_2_ increased to 400 μM, the neurites shortened or even disappeared, and the number was reduced (Figure 1c,d insert). These results demonstrate that H_2_O_2_ results in changes in the normal morphology of VSC4.1 cells.

### 3.3 H_2_O_2_ results in rearrangement of the actin cytoskeleton in VSC4.1 cells

**FIGURE 2:**
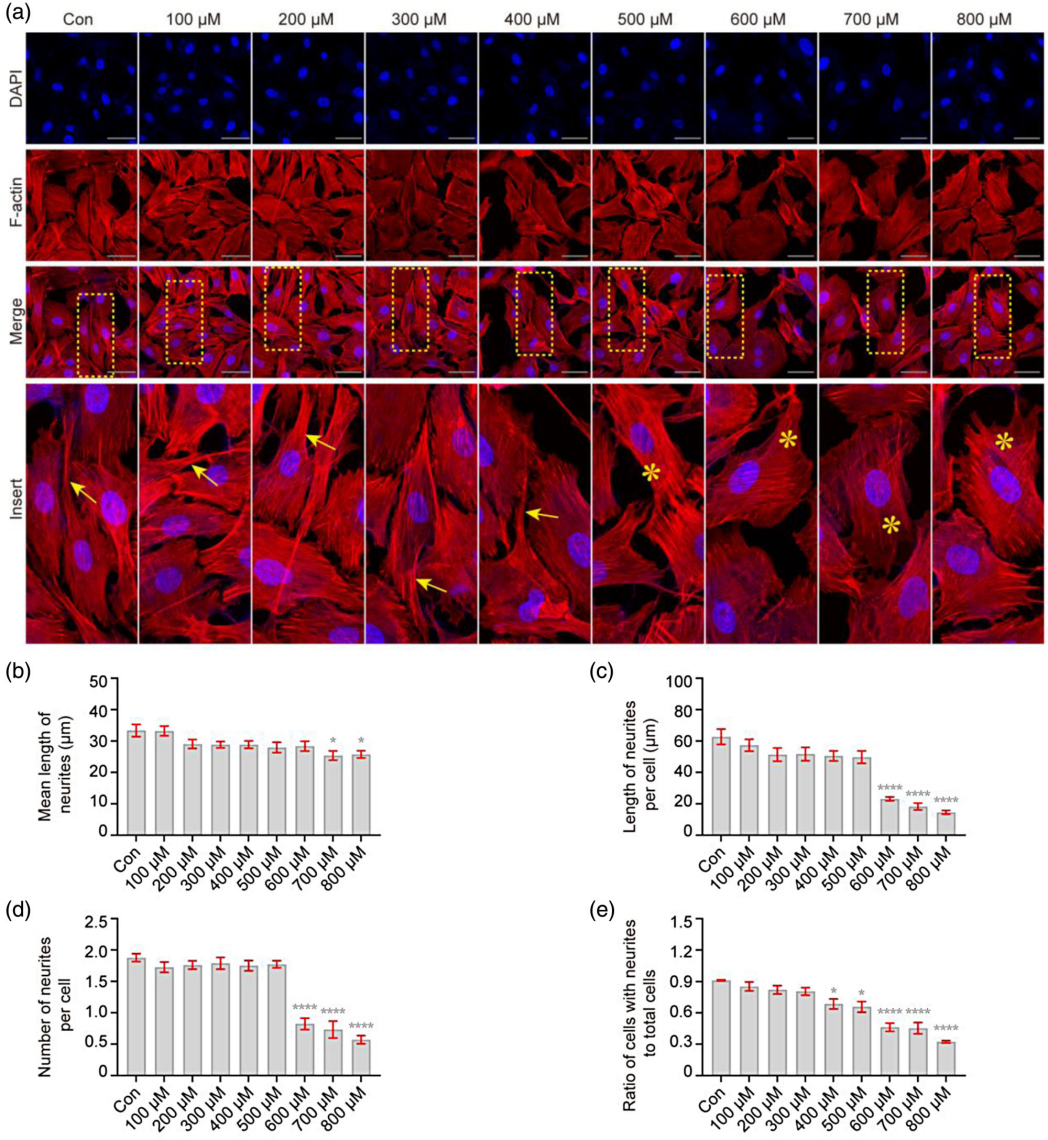
**H_2_O_2_ causes rearrangement of the actin cytoskeleton in VSC4.1 cells**. VSC4.1 cells were treated with different concentrations of H_2_O_2_ for 2 h, and changes in the actin cytoskeleton were detected by immunocytochemistry. (a) Representative images using confocal laser microscopy showed an rearrangement of the actin cytoskeleton in VSC4.1 cells with H_2_O_2_ treatment; phalloidin for F‐actin (red) and 4′,6‐diamidino‐2‐phenylindole (DAPI) (blue). Cellular neurites (yellow arrow →, normal; yellow asterisk *, exception). Scale bars: 50 μm. (b–e) As shown in quantitation of the length and number of neurites, shorter and fewer neurites were found in VSC4.1 cells treated with H_2_O_2_: mean length of neurites (b), length of neurites per cell (c), number of neurites per cell (d), and ratio of cells with neurites to total cells (e). One‐way analysis of variance (ANOVA) followed by Tukey's multiple comparisons test. Data are presented as mean ± standard error of mean (SEM). Compared with Con, *p < .05, ****p < .0001

The cytoskeleton of neurons is mainly composed of NFs, microfilaments, and microtubules, of which each structure performs important functions and forms a complex network through dynamic interaction, maintaining normal morphology and performing various cellular functions (Kennedy & Hanus, 2019). Among them, the actin cytoskeleton has a specific geometry that provides the cell with structural integrity and power (Pocaterra et al., 2019). Therefore, we determined actin cytoskeleton changes via phalloidin staining, observed with confocal microscopy. As shown in Figure 2a, the actin cytoskeleton in normal VSC4.1 cells was bundled with various networks; however, a dense arrangement was observed after the concentration of H_2_O_2_ increased to 500 μM, which was characterized by increased stress fiber formation; at the same time, characteristic neurites were found in normal VSC4.1 cells but became shorter (76%, *F* = 3.64, *p* < .05, Figure 2b; 37%, *F* = 27.95, *p* < .0001, part (c)) and reduced in number (44%, *F* = 41.00, *p* < .001, part (d); 67%, *F* = 18.44, *p* < .05, part (e)). These results suggest that H_2_O_2_ exposure causes a drastic rearrangement of the actin cytoskeleton in VSC4.1 cells.

### 3.4 H_2_O_2_ results in disordered microtubule formation in VSC4.1 cells

**FIGURE 3:**
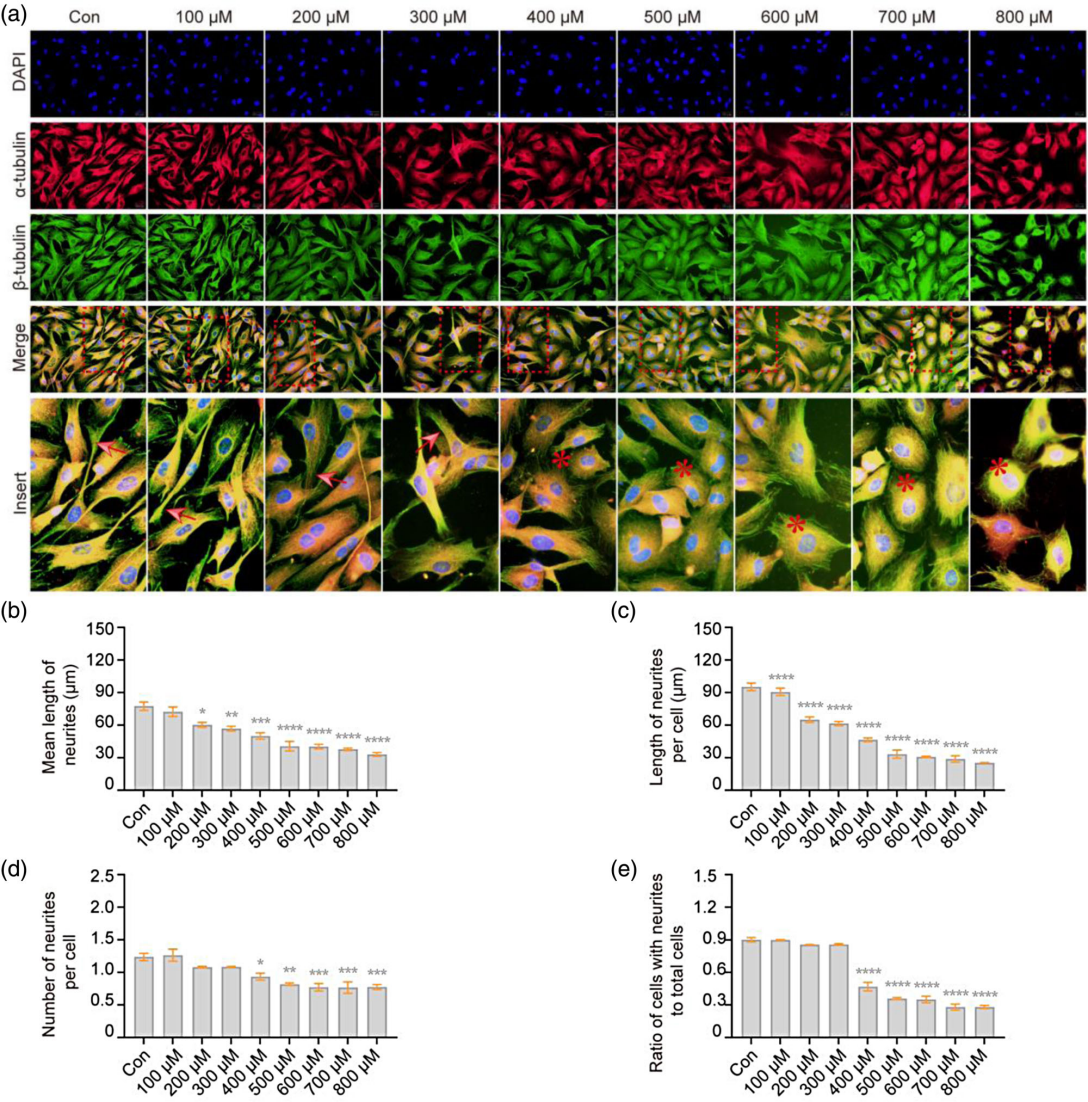
**H_2_O_2_ results in disordered microtubule structure in VSC4.1 cells**. VSC4.1 cells were treated with different concentrations of H_2_O_2_ for 2 h, and the changes in microtubules were detected by immunocytochemistry. (a) Representative images under a fluorescence microscope showed that the bundle structure of microtubules in normal VSC4.1 cells retracted after H_2_O_2_ treatment; α‐tubulin (red), β‐tubulin (green), and 4′,6‐diamidino‐2‐phenylindole (DAPI) (blue). Cellular neurites (red arrow →, normal; red asterisk *, exception). Scale bars: 50 μm. (b–e) As shown in quantitation of the length and number of neurites, shorter and fewer neurites were found in VSC4.1 cells treated with H_2_O_2_: mean length of neurites (b), length of neurites per cell (c), number of neurites per cell (d), and ratio of cells with neurites to total cells (e). One‐way analysis of variance (ANOVA) followed by Tukey's multiple comparisons test. Data are presented as mean ± standard error of mean (SEM). Compared with Con, *p < .05, **p < .01, ***p < .001, ****p < .0001

**FIGURE 4:**
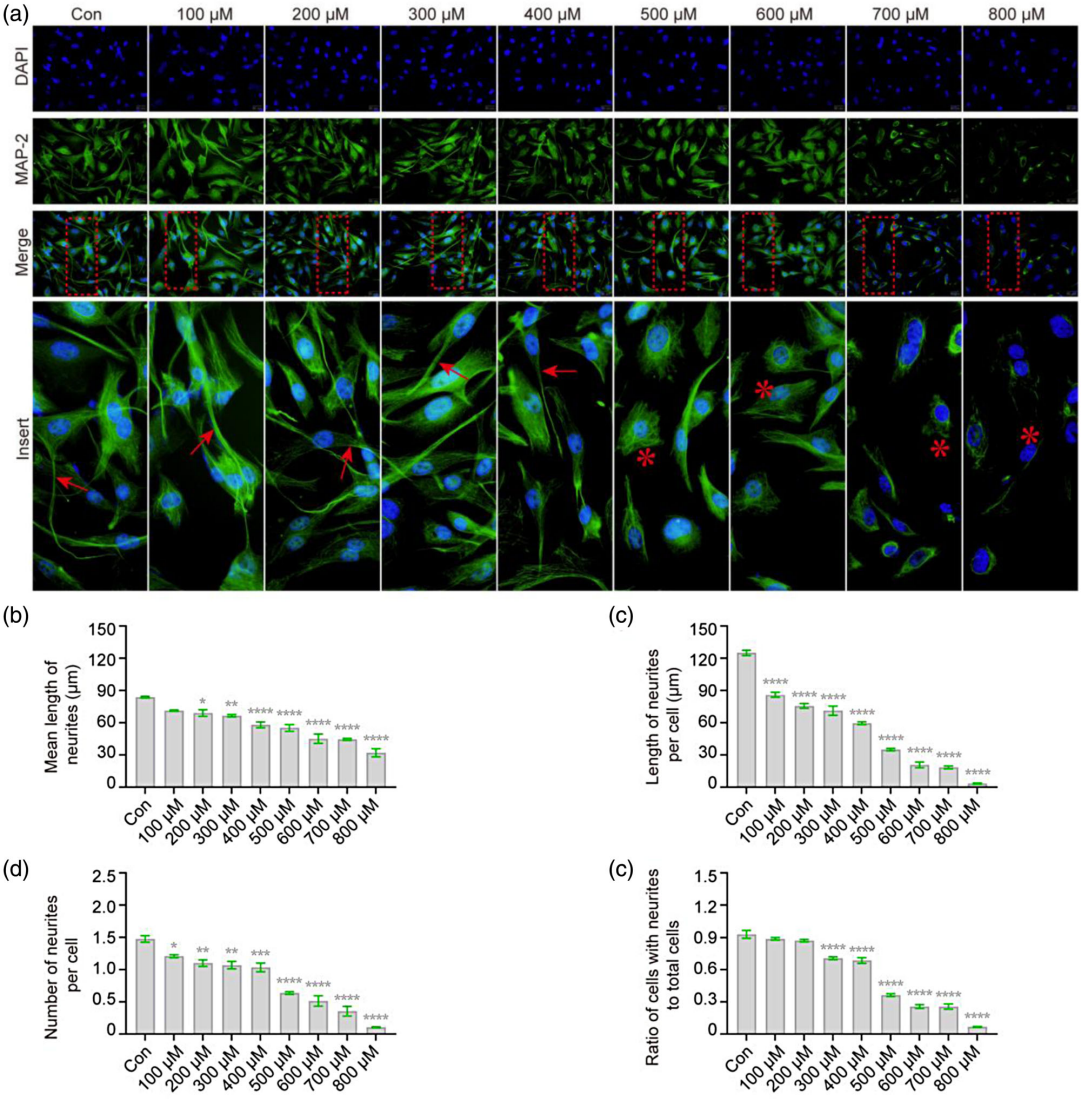
**H_2_O_2_ decreased the expression of MAP‐2 in VSC4.1 cells**. VSC4.1 cells were treated with different concentrations of H_2_O_2_ for 2 h, and the changes of microtubule‐associated protein 2 (MAP‐2) were detected by immunocytochemistry. (a) Representative images of VSC4.1 cells under a fluorescence microscope; MAP‐2 (green) and 4′,6‐diamidino‐2‐phenylindole (DAPI) (blue). Cellular neurites (red arrow →, normal; red asterisk *, exception). Scale bars: 50 μm. (b–e) As shown in quantitation of the length and number of neurites, shorter and fewer neurites were found in VSC4.1 cells treated with H_2_O_2_: mean length of neurites (b), length of neurites per cell (c), number of neurites per cell (d), and ratio of cells with neurites to total cells (e). One‐way analysis of variance (ANOVA) followed by Tukey's multiple comparisons test. Data are presented as mean ± standard error of mean (SEM). Compared with Con, *p < .05, **p < .01, ***p < .001, ****p < .0001

The highly specialized morphological characteristics of neurons show a unique dependence on microtubules (Kapitein & Hoogenraad, 2015). Therefore, we analyzed the effect of H_2_O_2_ exposure on microtubules in the cytoskeleton of VSC4.1 cells via α‐tubulin and β‐tubulin staining. The results show that, compared with the bundle structure in normal cells, microtubules were retracted after H_2_O_2_ treatment (Figure 3a), with a corresponding reduction in neurite length (78%, *F* = 27.75, *p* < .05, Figure 3b; 90%, *F* = 106.06, *p* < .0001, part (c)) and number (76%, *F* = 13.27, *p* < .05, part (d); 52%, *F* = 187.80, *p* < .0001, part (e)). MAP‐2, the most abundant microtubule‐associated protein, is associated not only with microtubule formation but also with actin in mature neurons (Dehmelt & Halpain, 2005). Therefore, we detected an expression of MAP‐2 in VSC4.1 cells after H_2_O_2_ exposure, and the results showed that the MAP‐2 immunoreactivity was reduced (Figure 4a). In addition, the decrease in MAP‐2 immunoreactivity coincided with a reduction in neurite length (82%, *F* = 37.29, *p* < .05, Figure 4b; 69%, *F* = 300.20, *p* < .0001, part (c)) and number (82%, *F* = 70.47, *p* < .05, part (d); 76%, *F* = 268.50, *p* < .0001, part (e)). In summary, H_2_O_2_ exposure leads to disordered microtubule formation in VSC4.1 cells.

### 3.5 H_2_O_2_ results in disordered neurofilament formation in VSC4.1 cells

**FIGURE 5:**
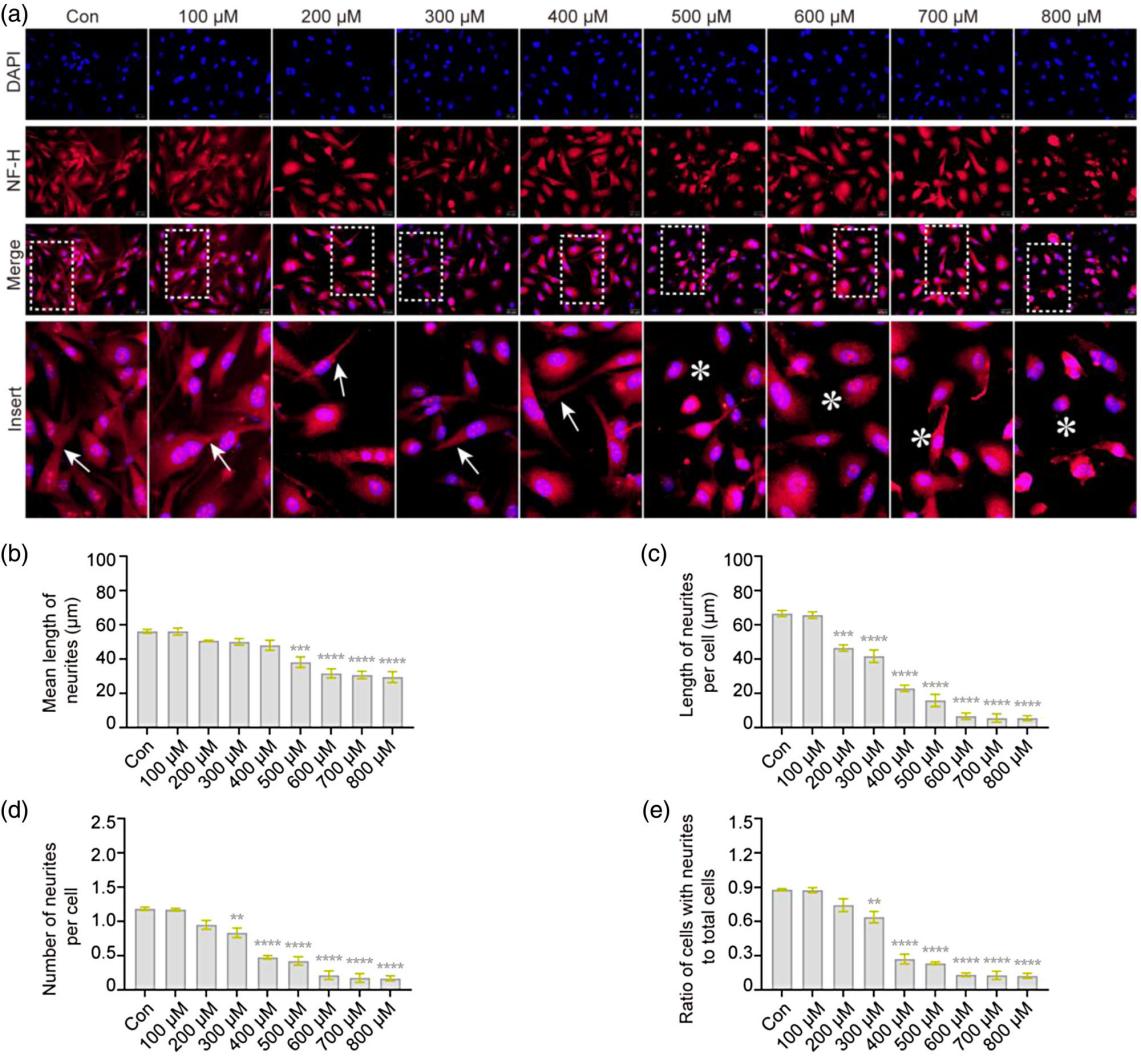
**H_2_O_2_ results in disordered of neurofilament structure in VSC4.1 cells**. VSC4.1 cells were treated with different concentrations of H_2_O_2_ for 2 h, and the changes in neurofilaments were detected by immunocytochemistry. (a) Representative images of VSC4.1 cells under a fluorescence microscope showed a nonfilamentous pattern of neurofilament heavy (NF‐H) and failing to extend to the neurite after H_2_O_2_ treatment; NF‐H (red) and 4′,6‐diamidino‐2‐phenylindole (DAPI) (blue). Cellular neurites (white arrow →, normal; white asterisk *, exception). Scale bars: 50 μm. (b–e) As shown in quantitation of the length and number of neurites, shorter and fewer neurites were found in VSC4.1 cells treated with H_2_O_2_: mean length of neurites (b), length of neurites per cell (c), number of neurites per cell (d), and ratio of cells with neurites to total cells (e). One‐way analysis of variance (ANOVA) followed by Tukey's multiple comparisons test. Data are presented as mean ± standard error of mean (SEM). Compared with Con, **p < .01, ***p < .001, ****p < .0001

NFs are the main intermediate filaments in the cytoskeleton of neurons and are important determinants of the shape and size of nerve cells (Gordon, 2020). To further clarify the changes in the cytoskeleton of spinal motor neurons under oxidative stress, we examined the effects of H_2_O_2_ exposure on NF‐H in VSC4.1 cells using immunofluorescence. After exposure to H_2_O_2_ at concentrations below 400 μM, no significant changes in NF‐H were observed compared with the normal group, which showed a normal filament structure; however, at concentrations from 500 μM, we observed that NF‐H was localized in the cell body or the proximal part of the neurite, appearing in a nonfilamentous pattern and failing to extend to the neurite (Figure 5a), coinciding with a reduction in neurite length (65%, *F* = 20.45, *p* < .001, Figure 5b; 70%, *F* = 112.00, *p* < .001, part (c)) and number (70%, *F* = 67.62, *p* < .01, part (d); 73%, *F* = 97.01, *p* < .01, part (e)). The results show that H_2_O_2_ destroys the normal structure of NFs in VSC4.1 cells.

### 3.6 H_2_O_2_ induces cell cycle arrest of VSC4.1 cells

**FIGURE 6:**
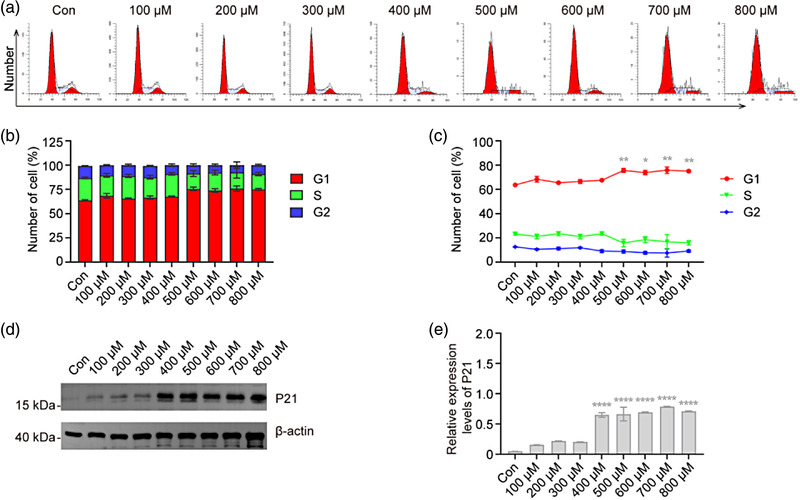
**H_2_O_2_ induces cycle arrest in VSC4.1 cells**. VSC4.1 cells were treated with H_2_O_2_ at the specified concentration for 2 h, and the cell cycle was analyzed. (a–c) VSC4.1 cells were sorted via flow cytometry and the cell cycle distribution was analyzed, the proportion of cells in the G0/G1 phase increased when the concentration of H_2_O_2_ reached 500 μM; Two‐way analysis of variance (ANOVA) followed by Tukey's multiple comparisons test. (d and e) Western blot and densitometric analysis for protein revealed a markedly increase of expression of cycle‐related protein P21 in VSC4.1 cells treated with H_2_O_2_. One‐way ANOVA followed by Tukey's multiple comparisons test. Data are expressed as mean ± standard error of mean (SEM). Compared with Con, *p < .05, **p < .01, ****p < .0001

CCK‐8 results in Figure 1a and b indicated that H_2_O_2_ exposure reduces the viability of VSC4.1 cells and indirectly suggested that H_2_O_2_ exposure inhibited VSC4.1 growth. We therefore determined the effects of H_2_O_2_ on the cell cycle by flow cytometry. We found that H_2_O_2_ exposure at concentrations between 100 and 400 μM had no significant effect on the cell cycle compared with the normal control group. By contrast, when the concentration reached 500 μM, the proportion of cells in the G0/G1 phase increased significantly, which was not the case for the S and G2/M phases (*p* < .05; Figure 6a–c). Next, we analyzed the expression of cell cycle‐related protein after H_2_O_2_ exposure and found an increasing trend of P21 protein expression level (*p* < .0001; Figure 6d,e). Taken together, these data indicate that H_2_O_2_ exposure results in G0/G1 phase arrest of VSC4.1 cells.

### 3.7 H_2_O_2_ induces apoptosis of VSC4.1 cells

**FIGURE 7:**
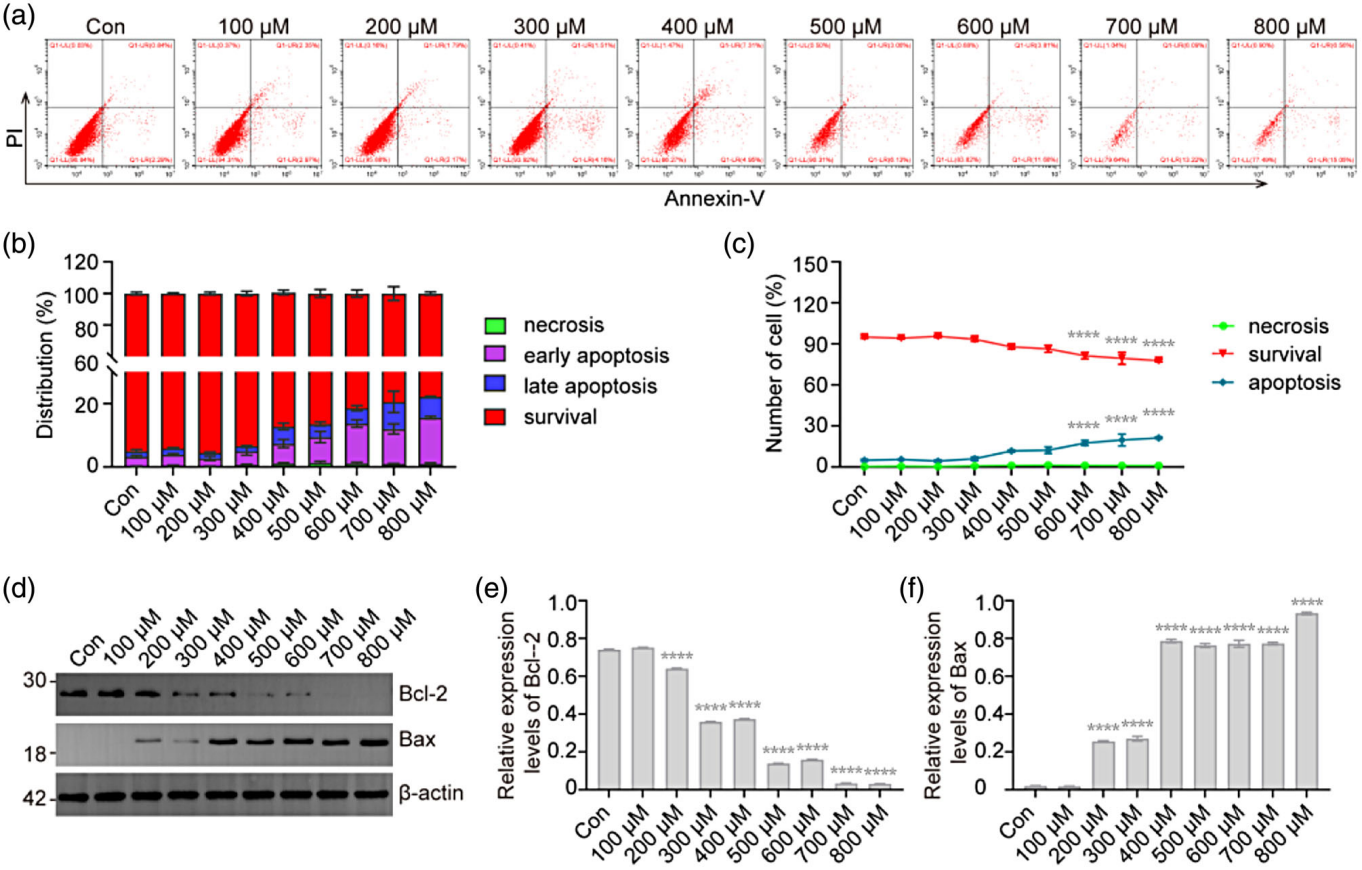
**H_2_O_2_ induces apoptosis of VSC4.1 cells**. VSC4.1 cells were treated with H_2_O_2_ for 2 h, and cell apoptosis was analyzed. (a–c) VSC4.1 cells were sorted via flow cytometry, and the apoptosis rate was quantified, H_2_O_2_ treatment (600, 700, and 800 μM) significantly increased the percentage of apoptotic cells; Two‐way analysis of variance (ANOVA) followed by Tukey's multiple comparisons test. (d–f) Results of western blot and densitometric analysis showed decreased and increased expression of apoptosis‐related genes *Bcl‐2* and *Bax* in VSC4.1 cells treated with H_2_O_2_. One‐way ANOVA followed by Tukey's multiple comparisons test. Data are expressed as mean ± standard error of mean (SEM). Compared with Con, ****p < .0001

Next, we investigated effect of H_2_O_2_ exposure on the apoptosis of VSC4.1 cells. After exposure to different concentrations of H_2_O_2_ for 2 h, we analyzed apoptosis via flow cytometry and found no significant differences in the rate of apoptosis with low levels of H_2_O_2_ exposure; however, at higher levels (600, 700, and 800 μM), significant increases in the percentage of apoptotic cells were observed (*p* < .0001, Figure 7a–c). These data suggest that H_2_O_2_ exposure induced the apoptosis of VSC4.1 cells. We further analyzed the expression of apoptosis‐related genes *Bax* and *Bcl‐2* (Nagata, 2018). Western blot results showed that the expression of Bcl‐2 decreased (*p* < .0001; Figure 7d,e), and that of Bax increased (*p* < .0001; Figure 7d,f). Taken together, the results indicate that H_2_O_2_ exposure induces apoptosis in VSC4.1 cells via regulation of Bcl‐2 and Bax.

### 3.8 H_2_O_2_ induces production of intracellular ROS in VSC4.1 cells

**FIGURE 8:**
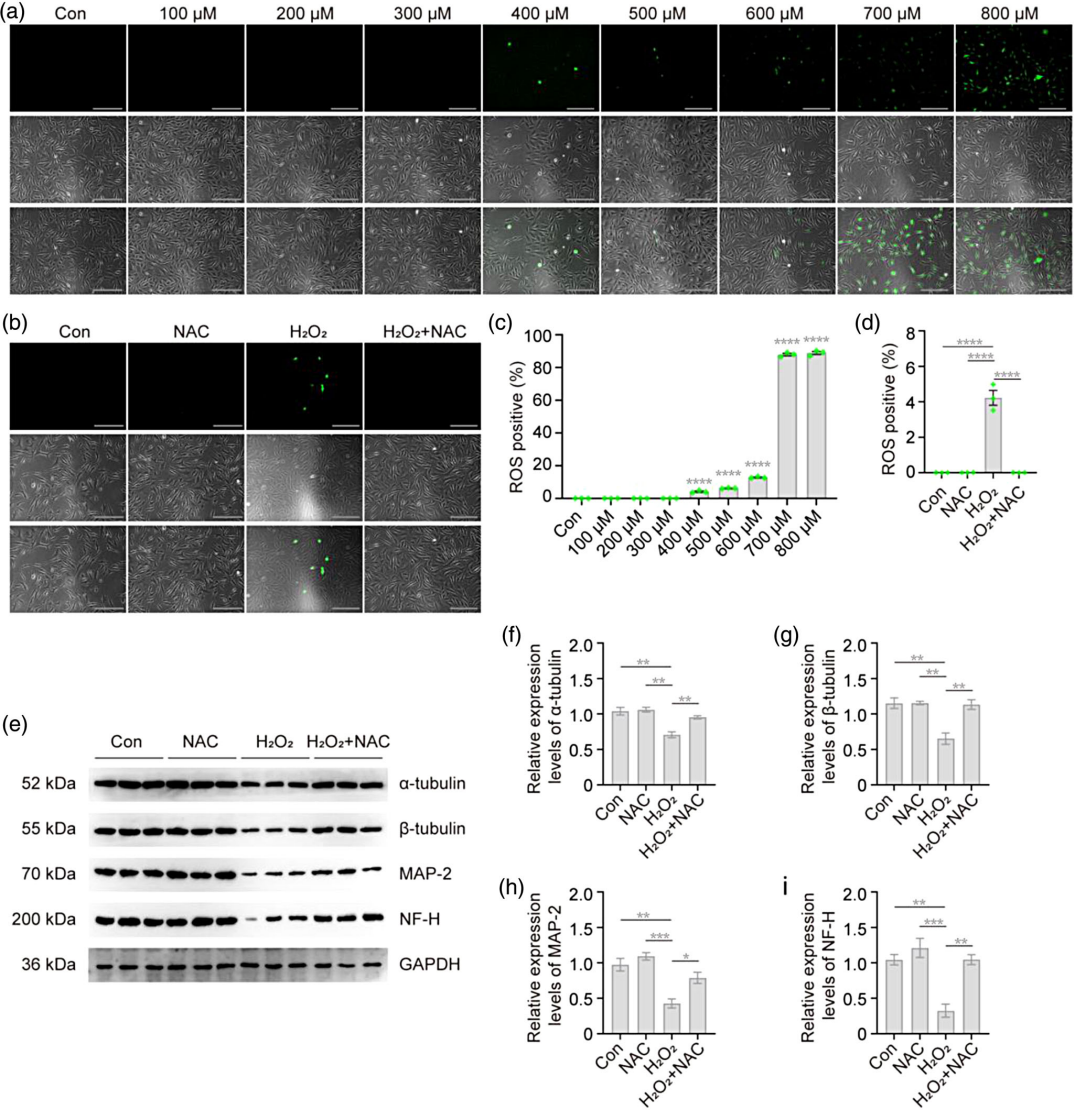
***N*‐acetyl‐l‐cysteine (NAC) attenuates the decreased expression of cytoskeletal proteins in VSC4.1 cells by H_2_O_2_**. (a and b) Representative images of VSC4.1 cells under fluorescence microscopy after reactive oxygen species (ROS) staining. ROS‐positive cells (green). Scale bars: 200 μm. VSC4.1 cells were treated with H_2_O_2_ for 2 h (a), or treated with or without NAC (2 mM) for 2 h after normal culture or H_2_O_2_ (500 μM) treatment for 2 h (b), after which ROS accumulation was analyzed. (c and d) Quantitation of the accumulation of ROS via the number of ROS‐positive cells showed that H_2_O_2_ increased intracellular ROS production in a dose‐dependent manner in VSC4.1 cells, which could be reversed by NAC. (c) One‐way analysis of variance (ANOVA) followed by Tukey's multiple comparisons test; compared with Con. (d) Two‐way ANOVA followed by Tukey's multiple comparisons test. (e–i) After normal culture or H_2_O_2_ (500 μM) treatment for 2 h, VSC4.1 cells were treated with or without NAC (2 mM) for 2 h. Western blot and densitometry of cytoskeletal analysis showed that NAC attenuated the decreased expression of cytoskeletal proteins in VSC4.1 cells treated with H_2_O_2_. Two‐way ANOVA followed by Tukey's multiple comparisons test. Data are expressed as mean ± standard error of mean (SEM). *p < .05, **p < .01, ***p < .001, ****p < .0001

To confirm whether exogenous H_2_O_2_ increases intracellular ROS production in VSC4.1 cells in vitro, we detected the changes in ROS after exposure to different doses of H_2_O_2_. At concentrations 400, 500, 600, 700, and 800 μM, increased intracellular ROS production was apparent (*p* < .0001; Figure 8a,c). In summary, our results suggest that H_2_O_2_ induces intracellular ROS production in a dose‐dependent manner in VSC4.1 cells.

### 3.9 NAC attenuates the decreased expression of cytoskeletal proteins in VSC4.1 cells by H_2_O_2_

NAC is the *N*‐acetyl derivative of cysteine and a general glutathione precursor (Dodd et al., 2008). Therefore, we determined whether the cytoskeleton disruption caused by H_2_O_2_ could be reversed by NAC. We observed that increased ROS levels were reversed by NAC (*p* < .0001; Figure 8b,d). Importantly, NAC treatment after exposure to H_2_O_2_ attenuated the decreased expression of cytoskeletal proteins (*p* < .001; Figure 8e–i). These results indicate that NAC reduces the production of ROS and alleviates the destruction of the cytoskeleton caused by oxidative stress induced by H_2_O_2_ in VSC4.1.

### 3.10 NAC promotes motor function recovery in mice with SCI

**FIGURE 9:**
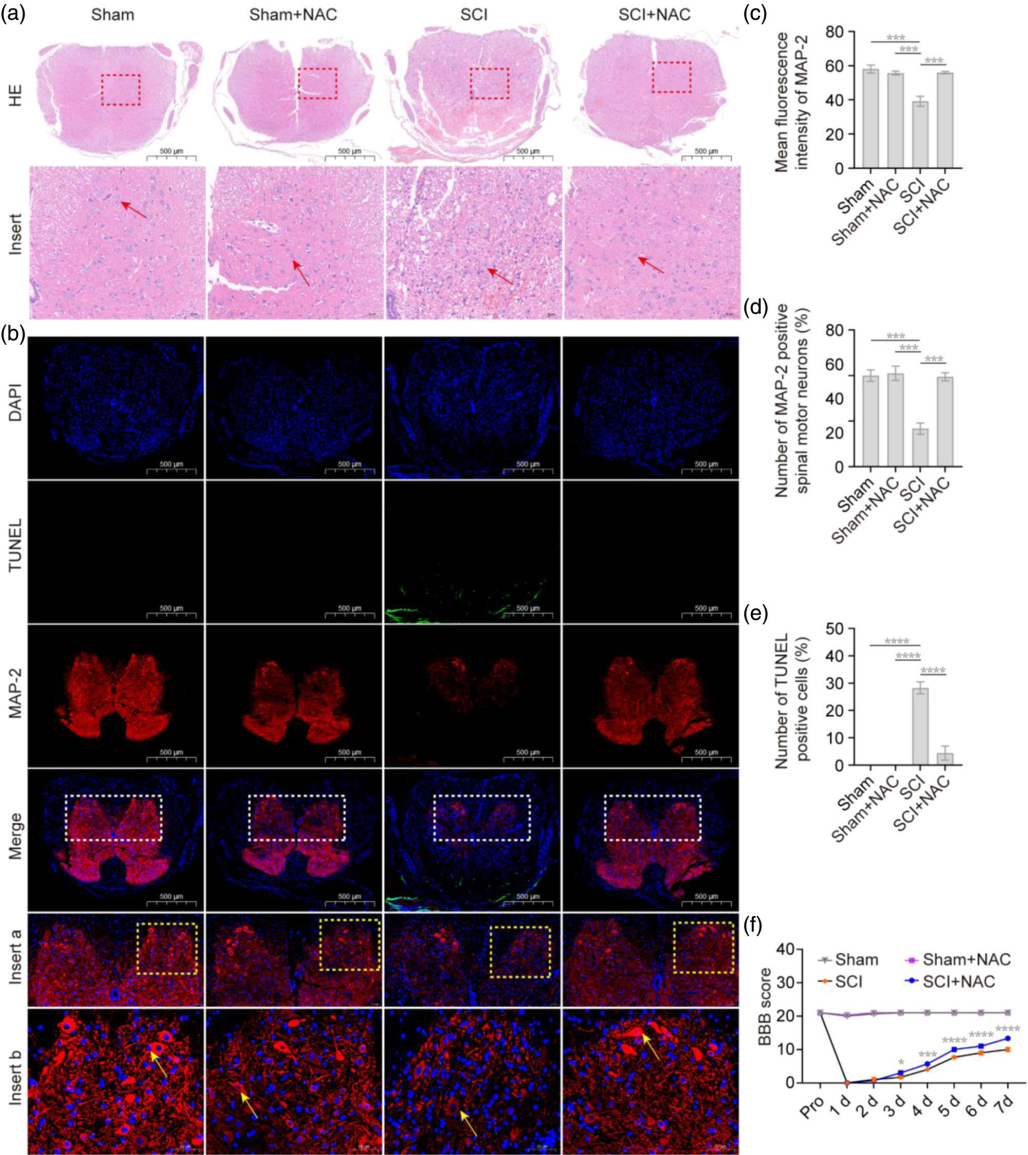
***N*‐acetyl‐l‐cysteine (NAC) promotes motor function recovery in mice with spinal cord injury (SCI)**. All experimental mice were divided into a sham operation group (Sham), SCI operation group (SCI), sham operation with NAC treatment group (Sham+NAC), and SCI operation with NAC treatment (SCI+NAC). Relevant experiments in vivo were performed on the third day after operation. (a) Representative images of hematoxylin and eosin staining of spinal cord tissue sections. Spinal motor neurons (red arrow →). Scale bars: 500 and 50 μm. (b) Representative images of MAP‐2 and transferase dUTP nick end labeling (TUNEL) staining immunofluorescence in spinal cord tissue sections; MAP‐2 (red), TUNEL (green), and 4′,6‐diamidino‐2‐phenylindole (DAPI) (blue). MAP‐2‐positive spinal motor neurons (yellow arrow →). Scale bars: 500 and 50 μm. (c–e) Quantitation analysis showed that NAC decreased the percentage of TUNEL‐positive cells in the spinal cord tissue and increased the mean fluorescence intensity of MAP‐2 in the spinal cord tissue and number of MAP‐2‐positive spinal motor neurons in the ventral horn of mice with SCI. (f) Basso, Beattie, and Bresnahan (BBB) scores showed that NAC promoted recovery of motor function after SCI in mice. Two‐way analysis of variance (ANOVA) followed by Tukey's multiple comparisons test. Data are expressed as mean ± standard error of mean (SEM). *p < .05, ***p < .0001, ****p < .0001

Based on the findings of cell experiments in vitro, we further investigated the effect of NAC on spinal motor neurons and motor ability recovery in mice with SCI in vivo. As shown in Figure 9a, spinal cord histopathology was evaluated by hematoxylin and eosin, with results indicating that the number of spinal motor neurons in the SCI group was significantly reduced at 3‐day post injury compared with the Sham group; however, there was a significant increase in the SCI+NAC group. Moreover, the results of MAP‐2 and TUNEL staining showed that the immunoreactivity of MAP‐2 and the number of MAP‐2‐positive spinal motor neurons in the ventral horn decreased, TUNEL‐positive cells increased of mice in the SCI group at 3 days after SCI, but all these changes of mice in the SCI+NAC group were significantly attenuated (*p* < .05; Figure 9b–e). Finally, we performed the BBB scoring post injury, with results indicating that the mice in the SCI group and SCI+NAC group were paralyzed immediately after surgery, with a BBB score of 0, indicating a successful establishment of the model. Interestingly, compared with that of the mice in SCI group, the BBB score of the mice in SCI+NAC group was significantly increased at 7 days after the operation (*p* < .05, Figure 9f). Taken together, these results suggest that NAC can reduce neuronal apoptosis and attenuate the decreased expression level of MAP‐2 in the spinal cord tissue and the loss of motor neurons in the ventral horn, promoting the recovery of hindlimb motor ability in mice with SCI at the early stage of injury.

## 4 DISCUSSION

Oxidative stress is a complex and crucial pathologic change of SCI, and the spinal motor neurons are more vulnerable to this damage. In this study, our results showed that oxidative stress induced by H_2_O_2_ exposure in vitro caused changes in VSC4.1 cell morphology, cycling, and apoptosis, and, in particular, the destruction of the cytoskeleton and a reduction in neurite length and number. Importantly, NAC attenuated the production of ROS and the destruction of cytoskeleton induced by H_2_O_2_ in VSC4.1 cells; moreover, NAC also enhanced the survival of spinal motor neurons and expression levels of MAP‐2, promoting locomotor recovery after SCI in mice.

The highly polarized morphology of neurons plays an important role in maintaining normal neuronal function. Therefore, for studies of oxidative damage in spinal motor neurons during and after SCI, the morphological changes are the primary focus of both the analysis and discussion outcome. Currently, few studies regarding the effects of oxidative stress on spinal motor neurons have been reported, in particular, on cytoskeleton. We found that H_2_O_2_ significantly altered the general morphology of VSC4.1 cells, especially in regard to neurite length and number. Notably, our results showed that H_2_O_2_ exposure caused significant changes in the expression levels of the cytoskeletal components, α‐tubulin, β‐tubulin, MAP‐2, and NF‐H in VSC4.1 cells, leading to a rearrangement of the actin cytoskeleton, disorder or absence of the microtubules and NFs, and a severe reduction in neurite length and number, indicating that oxidative stress leads to the destruction of the cytoskeleton of motor neurons in vitro.

On the one hand, in recent years, a growing number of studies have focused on the role of cytoskeleton in the nervous system. It has been reported that the stability and dynamics of the actin cytoskeleton and microtubules are key determinants of axonal degeneration after SCI (Mohan & John, 2015; Chalut & Paluch, 2016). Another study has shown that MAP‐2 expression in cell bodies and dendrites decreases rapidly and continuously in animal models of traumatic SCI (Yu et al., 2000). Larger neurons, such as spinal motor neurons, are easily affected by changes in NF status, which may lead to extensive disorders that are the cause and hallmarks of spinal motor neuron‐related diseases (Freeman et al., 2018). Spinal motor neurons are more susceptible to injury than other spinal cord neurons, and their loss has been observed in the early stages of SCI. On the other hand, in SCI, especially in the acute phase, there are few but definitive reports on changes in spinal motor neuron cell morphology (Bernstein et al., 1984; Cummings & Stelzner, 1988; Nacimiento et al., 1995; Grossman et al., 2001; Karalija et al., 2012). Taken together with these studies, our results suggest that oxidative stress, which plays a key role in the early stage of SCI, plays an important role in the disruption of the cytoskeleton of spinal motor neurons in SCI.

The above findings provide an experimental basis for further research regarding the effects and mechanisms of oxidative stress on cytoskeleton in spinal motor neurons. Of course, specific questions remain, such as are these changes merely a consequence of injury or are they also the cause of subsequent injuries during and after SCI? Further studies are needed to elucidate questions such as the one posed. Moreover, do the interactions between cytoskeletal components in spinal motor neurons play an important role in SCI? Our future studies will aim to explore and clarify these questions.

ROS generation plays a key role in the secondary pathophysiology of SCI and has been a research focus for at least a decade (Hall, 2011; Bains & Hall, 2012; Karalija et al., 2014; Coyoy‐Salgado et al., 2019; Zrzavy et al., 2021). NAC is a common antioxidant that can directly scavenge ROS. Some studies have shown that NAC has beneficial therapeutic effects on SCI (Karalija et al., 2012; Çavuş et al., 2014; Guo et al., 2015; Olakowska et al., 2017; Zhao et al., 2021). However, it is important to note that little information is available on the effects of antioxidant treatment on the cytoskeleton of spinal motor neurons. In our experiments, exogenous H_2_O_2_ reduced cell viability, leading to cell cycle arrest and apoptosis, which in turn triggers the production of intracellular ROS in VSC4.1 cells. Importantly, the increased ROS levels were reversed by the ROS scavenger NAC. Additionally, NAC treatment after exposure to H_2_O_2_ attenuated the decreased expression of cytoskeletal proteins in VSC4.1 cells. These data suggest that ROS signaling plays a key role in the disruption of cytoskeleton induced by oxidative stress in spinal motor neurons. Moreover, our results in vivo further showed that the increased number of TUNEL‐positive cells in the spinal cord tissue and the decreased number of MAP‐2‐positive motor neurons in the ventral horn were both reversed at 3 days after SCI in NAC‐treated mice. These data indicate that NAC can reduce apoptotic neurons, and importantly, it can also be used to promote the survival of spinal motor neurons following acute stress from SCI. Indeed, as reported, early intervention targeting the reduced neuronal survival induced by oxidative stress after SCI has important therapeutic value (Khan et al., 2011; Cheng et al., 2016; Rios et al., 2018; Gao et al., 2019; Zhang et al., 2021). More importantly, in our experiments, NAC increased the expression level of MAP‐2 at the injured spinal cord site and promoted the recovery of hindlimb motor function in mice with SCI at the early stage of injury. These findings suggest the potential of oxidative stress‐induced cytoskeleton repair to promote functional recovery after central nervous system injury. At the same time, the findings also emphasize the importance of further studying the effect and mechanism of oxidative stress on spinal motor neuron injury after SCI.

In conclusion, oxidative stress plays an important role in the cytoskeleton destruction of spinal motor neurons in SCI, and that the treatment of SCI on this basis is a promising strategy. These findings will help to elucidate the role of oxidative stress on spinal motor neuron injury in SCI and provide a theoretical basis for further studies on the cytoskeletal disruption of spinal motor neurons in SCI. Finally, this study provides references for further research on the pathology and underlying mechanism and treatment of SCI based on spinal motor neurons.

SCI repair and, therefore, subsequent patient therapy are difficult and face obstacles and challenges; thus, the selective regulation of the spinal motor neuronal cytoskeleton is emerging as a novel therapeutic strategy with great potential. However, which component should be targeted? Which treatments should be used and when should they be combined? All deserve further study. This may seem like a daunting task and challenge. Indeed, it is expected that exciting results will be found by combining the previous results that have been achieved by researchers regarding the understanding of phenotypes and key mechanisms involved in cytoskeleton damage caused by oxidative injury. Finally, it is expected that these results will lead to treatment that will contribute to the development of novel and effective comprehensive treatment regimens for SCI.

## CONFLICT OF INTEREST

The authors have no conflicts of interest.

### PEER REVIEW

The peer review history for this article is available at https://publons.com/publon/10.1002/brb3.2870.

## Data Availability

All data will be available upon reasonable request.

## References

[brb32870-bib-0001] Ahuja, C. S. , Wilson, J. R. , Nori, S. , Kotter, M. , Druschel, C. , Curt, A. , & Fehlings, M. G. (2017). Traumatic spinal cord injury. Nature Reviews. Disease Primers, 3, 17018. 10.1038/nrdp.2017.18 28447605

[brb32870-bib-0002] Azbill, R. D. , Mu, X. , Bruce‐Keller, A. J. , Mattson, M. P. , & Springer, J. E. (1997). Impaired mitochondrial function, oxidative stress and altered antioxidant enzyme activities following traumatic spinal cord injury. Brain Research, 765(2), 283–290. 10.1016/s0006-8993(97)00573-8 9313901

[brb32870-bib-0003] Bains, M. , & Hall, E. D. (2012). Antioxidant therapies in traumatic brain and spinal cord injury. Biochimica et biophysica acta, 1822(5), 675–684. 10.1016/j.bbadis.2011.10.017 22080976 PMC4134010

[brb32870-bib-0004] Basso, D. M. , Beattie, M. S. , & Bresnahan, J. C. (1995). A sensitive and reliable locomotor rating scale for open field testing in rats. Journal of Neurotrauma, 12(1), 1–21. 10.1089/neu.1995.12.1 7783230

[brb32870-bib-0005] Bernstein, J. J. , Wacker, W. , & Standler, N. (1984). Spinal motoneuron dendritic alteration after spinal cord hemisection in the rat. Experimental Neurology, 83(3), 548–554. 10.1016/0014-4886(84)90122-5 6698157

[brb32870-bib-0006] Cadotte, D. W. , & Fehlings, M. G. (2013). Spinal cord injury: Visualizing plasticity and repair in the injured CNS. Nature Reviews. Neurology, 9(10), 546–547. 10.1038/nrneurol.2013.190 24018480

[brb32870-bib-0007] Çavuş, U. Y. , Yılmaz, A. , Aytekin, M. N. , Taburcu, G. , Albayrak, A. , Yıldırım, S. , & Ağır, I. (2014). Efficacy of N‐acetylcysteine on neuroclinical, biochemical, and histopathological parameters in experimental spinal cord trauma: Comparison with methylprednisolone. European Journal of Trauma and Emergency Surgery: Official Publication of the European Trauma Society, 40(3), 363–371. 10.1007/s00068-013-0349-4 26816073

[brb32870-bib-0008] Chalut, K. J. , & Paluch, E. K. (2016). The actin cortex: A bridge between cell shape and function. Developmental Cell, 38(6), 571–573. 10.1016/j.devcel.2016.09.011 27676427

[brb32870-bib-0009] Cheng, P. , Kuang, F. , & Ju, G. (2016). Aescin reduces oxidative stress and provides neuroprotection in experimental traumatic spinal cord injury. Free Radical Biology & Medicine, 99, 405–417. 10.1016/j.freeradbiomed.2016.09.002 27596954

[brb32870-bib-0010] Coyoy‐Salgado, A. , Segura‐Uribe, J. J. , Guerra‐Araiza, C. , Orozco‐Suárez, S. , Salgado‐Ceballos, H. , Feria‐Romero, I. A. , Gallardo, J. M. , & Orozco‐Barrios, C. E. (2019). The importance of natural antioxidants in the treatment of spinal cord injury in animal models: An overview. Oxidative Medicine and Cellular Longevity, 2019, 3642491. 10.1155/2019/3642491 32676138 PMC7336207

[brb32870-bib-0011] Crawford, G. D., Jr , Le, W. D. , Smith, R. G. , Xie, W. J. , Stefani, E. , & Appel, S. H. (1992). A novel N18TG2 x mesencephalon cell hybrid expresses properties that suggest a dopaminergic cell line of substantia nigra origin. The Journal of Neuroscience: The Official Journal of the Society for Neuroscience, 12(9), 3392–3398. 10.1523/JNEUROSCI.12-09-03392.1992 1356145 PMC6575734

[brb32870-bib-0012] Cummings, J. P. , & Stelzner, D. J. (1988). Effect of spinal cord transection in the newborn, weanling, and adult rat on the morphology of thoracic motoneurons. Experimental Neurology, 100(2), 381–393. 10.1016/0014-4886(88)90116-1 3360076

[brb32870-bib-0013] David, G. , Mohammadi, S. , Martin, A. R. , Cohen‐Adad, J. , Weiskopf, N. , Thompson, A. , & Freund, P. (2019). Traumatic and nontraumatic spinal cord injury: Pathological insights from neuroimaging. Nature Reviews. Neurology, 15(12), 718–731. 10.1038/s41582-019-0270-5 31673093

[brb32870-bib-0014] Dehmelt, L. , & Halpain, S. (2005). The MAP2/Tau family of microtubule‐associated proteins. Genome Biology, 6(1), 204. 10.1186/gb-2004-6-1-204 15642108 PMC549057

[brb32870-bib-0015] Dodd, S. , Dean, O. , Copolov, D. L. , Malhi, G. S. , & Berk, M. (2008). N‐acetylcysteine for antioxidant therapy: Pharmacology and clinical utility. Expert Opinion on Biological Therapy, 8(12), 1955–1962. 10.1517/14728220802517901 18990082

[brb32870-bib-0016] Donato, A. , Kagias, K. , Zhang, Y. , & Hilliard, M. A. (2019). Neuronal sub‐compartmentalization: A strategy to optimize neuronal function. Biological Reviews of the Cambridge Philosophical Society, 94(3), 1023–1037. 10.1111/brv.12487 30609235 PMC6617802

[brb32870-bib-0017] El Masri, W. S. , & Kumar, N. (2011). Traumatic spinal cord injuries. Lancet (London, England), 377(9770), 972–974. 10.1016/S0140-6736(11)60248-1 21377200

[brb32870-bib-0018] Filli, L. , & Schwab, M. E. (2012). The rocky road to translation in spinal cord repair. Annals of Neurology, 72(4), 491–501. 10.1002/ana.23630 23109144

[brb32870-bib-0019] Fouad, K. , Popovich, P. G. , Kopp, M. A. , & Schwab, J. M. (2021). The neuroanatomical‐functional paradox in spinal cord injury. Nature Reviews. Neurology, 17(1), 53–62. 10.1038/s41582-020-00436-x 33311711 PMC9012488

[brb32870-bib-0020] Freeman, S. A. , Vega, A. , Riedl, M. , Collins, R. F. , Ostrowski, P. P. , Woods, E. C. , Bertozzi, C. R. , Tammi, M. I. , Lidke, D. S. , Johnson, P. , Mayor, S. , Jaqaman, K. , & Grinstein, S. (2018). Transmembrane pickets connect Cyto‐ and Pericellular skeletons forming barriers to receptor engagement. Cell, 172(1–2), 305–317.e10. 10.1016/j.cell.2017.12.023 29328918 PMC5929997

[brb32870-bib-0021] Gao, L. , Zhang, Z. , Xu, W. , Li, T. , Ying, G. , Qin, B. , Li, J. , Zheng, J. , Zhao, T. , Yan, F. , Zhu, Y. , & Chen, G. (2019). Natrium benzoate alleviates neuronal apoptosis via the DJ‐1‐Related Anti‐oxidative stress pathway involving Akt phosphorylation in a rat model of traumatic spinal cord injury. Frontiers in Molecular Neuroscience, 12, 42. 10.3389/fnmol.2019.00042 30853891 PMC6395451

[brb32870-bib-0022] GBD 2016 Neurology Collaborators . (2019). Global, regional, and national burden of neurological disorders, 1990–2016: A systematic analysis for the global burden of disease study 2016. The Lancet. Neurology, 18(5), 459–480. 10.1016/S1474-4422(18)30499-X 30879893 PMC6459001

[brb32870-bib-0023] Gordon, B. A. (2020). Neurofilaments in disease: What do we know? Current Opinion in Neurobiology, 61, 105–115. 10.1016/j.conb.2020.02.001 32151970 PMC7198337

[brb32870-bib-0024] Grossman, S. D. , Rosenberg, L. J. , & Wrathall, J. R. (2001). Temporal‐spatial pattern of acute neuronal and glial loss after spinal cord contusion. Experimental Neurology, 168(2), 273–282. 10.1006/exnr.2001.7628 11259115

[brb32870-bib-0025] Grumbles, R. M. , & Thomas, C. K. (2017). Motoneuron death after human spinal cord injury. Journal of Neurotrauma, 34(3), 581–590. 10.1089/neu.2015.4374 27349409 PMC5286554

[brb32870-bib-0026] Guo, J. , Li, Y. , Chen, Z. , He, Z. , Zhang, B. , Li, Y. , Hu, J. , Han, M. , Xu, Y. , & Li, Y. (2015). N‐acetylcysteine treatment following spinal cord trauma reduces neural tissue damage and improves locomotor function in mice. Molecular Medicine Reports, 12(1), 37–44. 10.3892/mmr.2015.3390 25738883 PMC4438879

[brb32870-bib-0027] Hall, E. D. (2011). Antioxidant therapies for acute spinal cord injury. Neurotherapeutics: The Journal of the American Society for Experimental Neurotherapeutics, 8(2), 152–167. 10.1007/s13311-011-0026-4 21424941 PMC3101837

[brb32870-bib-0028] Hammond, D. N. , Wainer, B. H. , Tonsgard, J. H. , & Heller, A. (1986). Neuronal properties of clonal hybrid cell lines derived from central cholinergic neurons. Science (New York, N.Y.), 234(4781), 1237–1240. 10.1126/science.3775382 3775382

[brb32870-bib-0029] Hejrati, N. , & Fehlings, M. G. (2021). A review of emerging neuroprotective and neuroregenerative therapies in traumatic spinal cord injury. Current Opinion in Pharmacology, 60, 331–340. 10.1016/j.coph.2021.08.009 34520943

[brb32870-bib-0030] Kapitein, L. C. , & Hoogenraad, C. C. (2015). Building the neuronal microtubule cytoskeleton. Neuron, 87(3), 492–506. 10.1016/j.neuron.2015.05.046 26247859

[brb32870-bib-0031] Karalija, A. , Novikova, L. N. , Kingham, P. J. , Wiberg, M. , & Novikov, L. N. (2012). Neuroprotective effects of N‐acetyl‐cysteine and acetyl‐L‐carnitine after spinal cord injury in adult rats. PLoS One, 7(7), e41086. 10.1371/journal.pone.0041086 22815926 PMC3398872

[brb32870-bib-0032] Karalija, A. , Novikova, L. N. , Kingham, P. J. , Wiberg, M. , & Novikov, L. N. (2014). The effects of N‐acetyl‐cysteine and acetyl‐L‐carnitine on neural survival, neuroinflammation and regeneration following spinal cord injury. Neuroscience, 269, 143–151. 10.1016/j.neuroscience.2014.03.042 24680856

[brb32870-bib-0033] Kennedy, M. J. , & Hanus, C. (2019). Architecture and dynamics of the neuronal secretory network. Annual Review of Cell and Developmental Biology, 35, 543–566. 10.1146/annurev-cellbio-100818-125418 PMC6935261 31283381

[brb32870-bib-0034] Khan, M. , Sakakima, H. , Dhammu, T. S. , Shunmugavel, A. , Im, Y. B. , Gilg, A. G. , Singh, A. K. , & Singh, I. (2011). S‐nitrosoglutathione reduces oxidative injury and promotes mechanisms of neurorepair following traumatic brain injury in rats. Journal of Neuroinflammation, 8, 78. 10.1186/1742-2094-8-78 21733162 PMC3158546

[brb32870-bib-0035] Lee, B. B. , Cripps, R. A. , Fitzharris, M. , & Wing, P. C. (2014). The global map for traumatic spinal cord injury epidemiology: Update 2011, global incidence rate. Spinal Cord, 52(2), 110–116. 10.1038/sc.2012.158 23439068

[brb32870-bib-0036] Li, H. , Wang, C. , He, T. , Zhao, T. , Chen, Y. Y. , Shen, Y. L. , Zhang, X. , & Wang, L. L. (2019). Mitochondrial transfer from bone marrow mesenchymal stem cells to motor neurons in spinal cord injury rats via gap junction. Theranostics, 9(7), 2017–2035. 10.7150/thno.29400 31037154 PMC6485285

[brb32870-bib-0037] Mohan, R. , & John, A. (2015). Microtubule‐associated proteins as direct crosslinkers of actin filaments and microtubules. IUBMB Life, 67(6), 395–403. 10.1002/iub.1384 26104829

[brb32870-bib-0038] Nacimiento, W. , Sappok, T. , Brook, G. A. , Tóth, L. , Schoen, S. W. , Noth, J. , & Kreutzberg, G. W. (1995). Structural changes of anterior horn neurons and their synaptic input caudal to a low thoracic spinal cord hemisection in the adult rat: A light and electron microscopic study. Acta Neuropathologica, 90(6), 552–564. 10.1007/BF00318567 8615075

[brb32870-bib-0039] Nagata, S. (2018). Apoptosis and clearance of apoptotic cells. Annual Review of Immunology, 36, 489–517. 10.1146/annurev-immunol-042617-053010 29400998

[brb32870-bib-0040] Olakowska, E. , Marcol, W. , Właszczuk, A. , Woszczycka‐Korczyńska, I. , & Lewin‐Kowalik, J. (2017). The neuroprotective effect of N‐acetylcysteine in spinal cord‐injured rats. Advances in Clinical and Experimental Medicine: Official Organ Wroclaw Medical University, 26(9), 1329–1334. 10.17219/acem/65478 29442452

[brb32870-bib-0041] Pocaterra, A. , Santinon, G. , Romani, P. , Brian, I. , Dimitracopoulos, A. , Ghisleni, A. , Carnicer‐Lombarte, A. , Forcato, M. , Braghetta, P. , Montagner, M. , Galuppini, F. , Aragona, M. , Pennelli, G. , Bicciato, S. , Gauthier, N. , Franze, K. , & Dupont, S. (2019). F‐actin dynamics regulates mammalian organ growth and cell fate maintenance. Journal of Hepatology, 71(1), 130–142. 10.1016/j.jhep.2019.02.022 30878582

[brb32870-bib-0042] Rios, C. , Santander, I. , Méndez‐Armenta, M. , Nava‐Ruiz, C. , Orozco‐Suárez, S. , Islas, M. , Barón‐Flores, V. , & Diaz‐Ruiz, A. (2018). Metallothionein‐I + II reduces oxidative damage and apoptosis after traumatic spinal cord injury in rats. Oxidative Medicine and Cellular Longevity, 2018, 3265918. 10.1155/2018/3265918 30524652 PMC6247576

[brb32870-bib-0043] Rubiano, A. M. , Carney, N. , Chesnut, R. , & Puyana, J. C. (2015). Global neurotrauma research challenges and opportunities. Nature, 527(7578), S193–S197. 10.1038/nature16035 26580327

[brb32870-bib-0044] Selvarajah, S. , Hammond, E. R. , & Schneider, E. B. (2015). Trends in traumatic spinal cord injury. JAMA, 314(15), 1643. 10.1001/jama.2015.11194 26501541

[brb32870-bib-0045] Sies, H. , & Jones, D. P. (2020). Reactive oxygen species (ROS) as pleiotropic physiological signalling agents. Nature Reviews. Molecular Cell Biology, 21(7), 363–383. 10.1038/s41580-020-0230-3 32231263

[brb32870-bib-0046] Smith, R. G. , Alexianu, M. E. , Crawford, G. , Nyormoi, O. , Stefani, E. , & Appel, S. H. (1994). Cytotoxicity of immunoglobulins from amyotrophic lateral sclerosis patients on a hybrid motoneuron cell line. Proceedings of the National Academy of Sciences of the United States of America, 91(8), 3393–3397. 10.1073/pnas.91.8.3393 8159758 PMC43583

[brb32870-bib-0047] Sofroniew, M. V. (2018). Dissecting spinal cord regeneration. Nature, 557(7705), 343–350. 10.1038/s41586-018-0068-4 29769671

[brb32870-bib-0048] Squair, J. W. , Gautier, M. , Sofroniew, M. V. , Courtine, G. , & Anderson, M. A. (2021). Engineering spinal cord repair. Current Opinion in Biotechnology, 72, 48–53. 10.1016/j.copbio.2021.10.006 34695766

[brb32870-bib-0049] von Leden, R. E. , Yauger, Y. J. , Khayrullina, G. , & Byrnes, K. R. (2017). Central nervous system injury and nicotinamide adenine dinucleotide phosphate oxidase: Oxidative stress and therapeutic targets. Journal of Neurotrauma, 34(4), 755–764. 10.1089/neu.2016.4486 27267366 PMC5335782

[brb32870-bib-0050] Wang, F. , Chang, S. , Li, J. , Wang, D. , Li, H. , & He, X. (2021). Lithium alleviated spinal cord injury (SCI)‐induced apoptosis and inflammation in rats via BDNF‐AS/miR‐9‐5p axis. Cell and Tissue Research, 384(2), 301–312. 10.1007/s00441-020-03298-3 33464390

[brb32870-bib-0051] Xu, W. , Chi, L. , Xu, R. , Ke, Y. , Luo, C. , Cai, J. , Qiu, M. , Gozal, D. , & Liu, R. (2005). Increased production of reactive oxygen species contributes to motor neuron death in a compression mouse model of spinal cord injury. Spinal Cord, 43(4), 204–213. 10.1038/sj.sc.3101674 15520836

[brb32870-bib-0052] Yu, W. R. , Westergren, H. , Farooque, M. , Holtz, A. , & Olsson, Y. (2000). Systemic hypothermia following spinal cord compression injury in the rat: An immunohistochemical study on MAP 2 with special reference to dendrite changes. Acta Neuropathologica, 100(5), 546–552. 10.1007/s004010000206 11045677

[brb32870-bib-0053] Zawadzka, M. , Kwaśniewska, A. , Miazga, K. , & Sławińska, U. (2021). Perspectives in the cell‐based therapies of various aspects of the spinal cord injury‐associated pathologies: Lessons from the animal models. Cells, 10(11), 2995. 10.3390/cells10112995 34831217 PMC8616284

[brb32870-bib-0054] Zhang, J. , Li, Y. , Xiong, J. , Xu, H. , Xiang, G. , Fan, M. , Zhou, K. , Lin, Y. , Chen, X. , Xie, L. , Zhang, H. , Wang, J. , & Xiao, J. (2021). Delivery of pOXR1 through an injectable liposomal nanoparticle enhances spinal cord injury regeneration by alleviating oxidative stress. Bioactive Materials, 6(10), 3177–3191. 10.1016/j.bioactmat.2021.03.001 33778197 PMC7970014

[brb32870-bib-0055] Zhao, X. , Zhao, X. , & Wang, Z. (2021). Synergistic neuroprotective effects of hyperbaric oxygen and N‐acetylcysteine against traumatic spinal cord injury in rat. Journal of Chemical Neuroanatomy, 118, 102037. 10.1016/j.jchemneu.2021.102037 34601074

[brb32870-bib-0056] Zrzavy, T. , Schwaiger, C. , Wimmer, I. , Berger, T. , Bauer, J. , Butovsky, O. , Schwab, J. M. , Lassmann, H. , & Höftberger, R. (2021). Acute and non‐resolving inflammation associate with oxidative injury after human spinal cord injury. Brain: A Journal of Neurology, 144(1), 144–161. 10.1093/brain/awaa360 33578421 PMC7880675

